# Preoperative prediction of peripancreatic vein invasion by pancreatic head cancer

**DOI:** 10.1186/s40644-018-0179-z

**Published:** 2018-12-10

**Authors:** Yi-Nan Shen, Cheng-Xiang Guo, Yao Pan, Yi-Wen Chen, Tian-Yu Tang, Yu-Wei Li, Jun-Hua Lu, Gang Jin, Ren-Yi Qin, Wei-Yun Yao, Ting-Bo Liang, Xue-Li Bai

**Affiliations:** 1 Department of Hepatobiliary and Pancreatic Surgery, the Second Affiliated Hospital, Zhejiang University School of Medicine, 88 Jiefang Road, Hangzhou, 310009 Zhejiang Province China; 2 Zhejiang Provincial Key Laboratory of Pancreatic Disease, Hangzhou, China; 3 Department of Radiology, the Second Affiliated Hospital, Zhejiang University School of Medicine, Hangzhou, China; 4 The 5th Department of Hepatic Surgery, Eastern Hepatobiliary Surgery Hospital, the Second Military Medical University, Shanghai, China; 5 Department of General Surgery, Changhai Hospital, the Second Military Medical University, Shanghai, China; 6 Department of Pancreatic-Biliary Surgery, Tongji Hospital, Tongji Medical College, Huazhong University of Science and Technology, Wuhan, China; 7 Department of General Surgery, The People’s Hospital of Changxing County, Huzhou, China

**Keywords:** Pancreatic neoplasms, Pancreaticoduodenectomy, Risk factors

## Abstract

**Background:**

Pancreatic adenocarcinoma is often diagnosed at an advanced stage when adjacent vascular invasion is present. Accurate evaluation of presence of vascular invasion can help guide therapy. The aim of this study was to construct a nomogram for preoperative prediction of peripancreatic vein invasion in patients with pancreatic head cancer.

**Study design:**

Data of patients with carcinoma head of pancreas and suspected peripancreatic invasion (*n* = 247) who underwent pancreatic resection with venous reconstruction between January 2012 and January 2017 at four academic institutions were retrospectively analyzed. Univariate and multivariate analyses were used to identify independent risk factors for vein invasion from among demographic, biological, conditional host-related, and anatomical data. A predictive nomogram was constructed based on the identified independent risk factors.

**Results:**

The nomogram was constructed using data from 181 patients while the validation cohort consisted of 66 patients. Length of tumor contact (*P* = 0.031), circumferential vein involvement (*P* = 0.048), and venous contour abnormalities (*P* = 0.001) were independent predictors of venous invasion. The C-index of the model in predicting venous invasion was 0.963 for the external validation cohort. Patients could be assigned into low- (< 50%), intermediate- (50–90%), and high-risk (> 90%) groups based on the nomogram to facilitate personalized management.

**Conclusions:**

Vein invasion by pancreatic head cancer is mainly associated with anatomical factors. The nomogram for prediction of vein invasion was found to be practicable.

**Electronic supplementary material:**

The online version of this article (10.1186/s40644-018-0179-z) contains supplementary material, which is available to authorized users.

## Introduction

Pancreatic cancer is a lethal disease with high morbidity and dismal prognosis [1, 2]. In contrast to the steady improvement in survival rates in other cancers, the survival rate in pancreatic cancer remains low at 8%. This poor survival rate is partly because more than one-half of cases are diagnosed at an advanced stage, when the 5-year survival is only 3% [2]. Although a meta-analysis as reported that 20% of pancreatic cancer patients are eligible for one-stage resection by imaging [3], in fact 14–30% of these patients will be found to be unsuitable for resection during surgery [4]. Peripancreatic vascular invasion, which has major impact on prognosis, is an important criterion to consider when assessing resectability [5–7]. For patients with peripancreatic venous involvement—i.e., of the portal vein (PV) or superior mesenteric vein (SMV)—pancreaticoduodenectomy with PV/SMV resection and reconstruction (PSRR) substantially increases the probability of achieving R0 resection [8–10]. However, according to some reports 35–60% of pancreatic cancer patients undergoing PSRR have no histological evidence of PV/SMV invasion [11–14]. In addition, a meta-analysis has shown increased postoperative mortality, high percentage of patients couldn’t get R0 resection, and worse survival after pancreatic resection with PSRR [15]. Accurate preoperative evaluation of the presence of vein invasion is therefore essential, as it can help avoid unnecessary PSRR. In addition, patients without vein invasion also need to be correctly identified so that neoadjuvant therapy (NAT) is not administered. Unnecessary NAT could lead to resistance to treatment and tumor progression and, in some cases, dangerously delay surgical resection.

Several models have been proposed for predicting prognosis in individual patients and for guiding therapy [16–22], but none have found wide acceptance. For instance, with Klauss’s criteria, which are based on computed tomography (CT) findings, it is difficult to tell whether vessel compression is by a benign or malignant lesion [21, 23]. Another model that was proposed by Teramura et al. has good sensitivity and negative predictive value (97.6 and 97.5%, respectively) but is limited by poor specificity and positive predictive value (60 and 61.2%, respectively) [20]. Furthermore, some of the proposed models were based on relatively small cohorts [22].

Nomograms have proved to be useful tools for predicting risk of adverse events and likelihood of survival in various clinical scenarios [24–26], and are an effective substitute for the traditional TNM staging system for predicting survival in many cancers [27]. Compared to other decision aids such as risk groupings, probability tables, artificial neural networks, and classification and regression tree analyses, nomograms can provide evidence-based and highly accurate risk estimates for individualized decision-making [28]. Furthermore, nomograms are visual tools and easily applied in the clinic.

The aim of this retrospective study was to construct a nomogram for preoperative prediction of peripancreatic vein invasion in patients with pancreatic head cancer.

## Materials and methods

### Patients

Patients with adenocarcinoma of the head of pancreas and suspected peripancreatic vein invasion who underwent attempted curative pancreatic resection and venous reconstruction between January 2012 and January 2017 at one of four academic institutions in China (Second Affiliated Hospital of Zhejiang University School of Medicine, Hangzhou; Tongji Hospital, Wuhan; Eastern Hepatobiliary Surgery Hospital, Shanghai; and Changhai Hospital, Shanghai) were eligible for inclusion in this study. These four institutions are high-volume centers for pancreatic cancer surgery. Only patients who underwent pancreaticoduodenectomy (R0 or R1 resection) [29] and had histopathologically confirmed pancreatic head cancer were included. Patients were excluded if they 1) had history of any other malignant tumor; 2) had received NAT; 3) had undergone any surgical procedure other than complete resection of macroscopic pancreatic tumor and venous reconstruction; 4) had died within 90 days of surgery; or 5) preoperative radiological imaging revealed arterial invasion by tumor. Since all our patients were classified as T3 [30], with no distant metastasis (with the possible exception of lymph node metastasis), only lymph node staging on preoperative radiological imaging was included in the analysis.

All patients were followed-up, first, at the end of the first month after surgery, then once every 3 months for the first 2 years, and thereafter once every 6 months. At each follow-up visit, detailed clinical history was recorded and a complete physical examination performed. CT scans were carried out once every 6 months (or more often if clinically indicated). This study was approved by the ethics committees of all involved hospitals. Written informed consent was obtained from patients for use of their clinical data for research.

### Included factors

The included parameters were selected from three distinct dimensions (anatomical, biological, and conditional) according to an international consensus borderline resectable pancreatic ductal adenocarcinoma (BR-PDAC) [31]:

- Anatomical factors: length of tumor contact, circumferential vein involvement, venous contour abnormalities and type of vessels involvement;
- Biological factors: CA19–9, total bilirubin (TB) and Albumin
- Conditional factors: activities of daily living (ADL), jaundice, pain, weight loss, lymph node staging and tumor size

### Imaging analysis and examination techniques

**Fig. 1:**
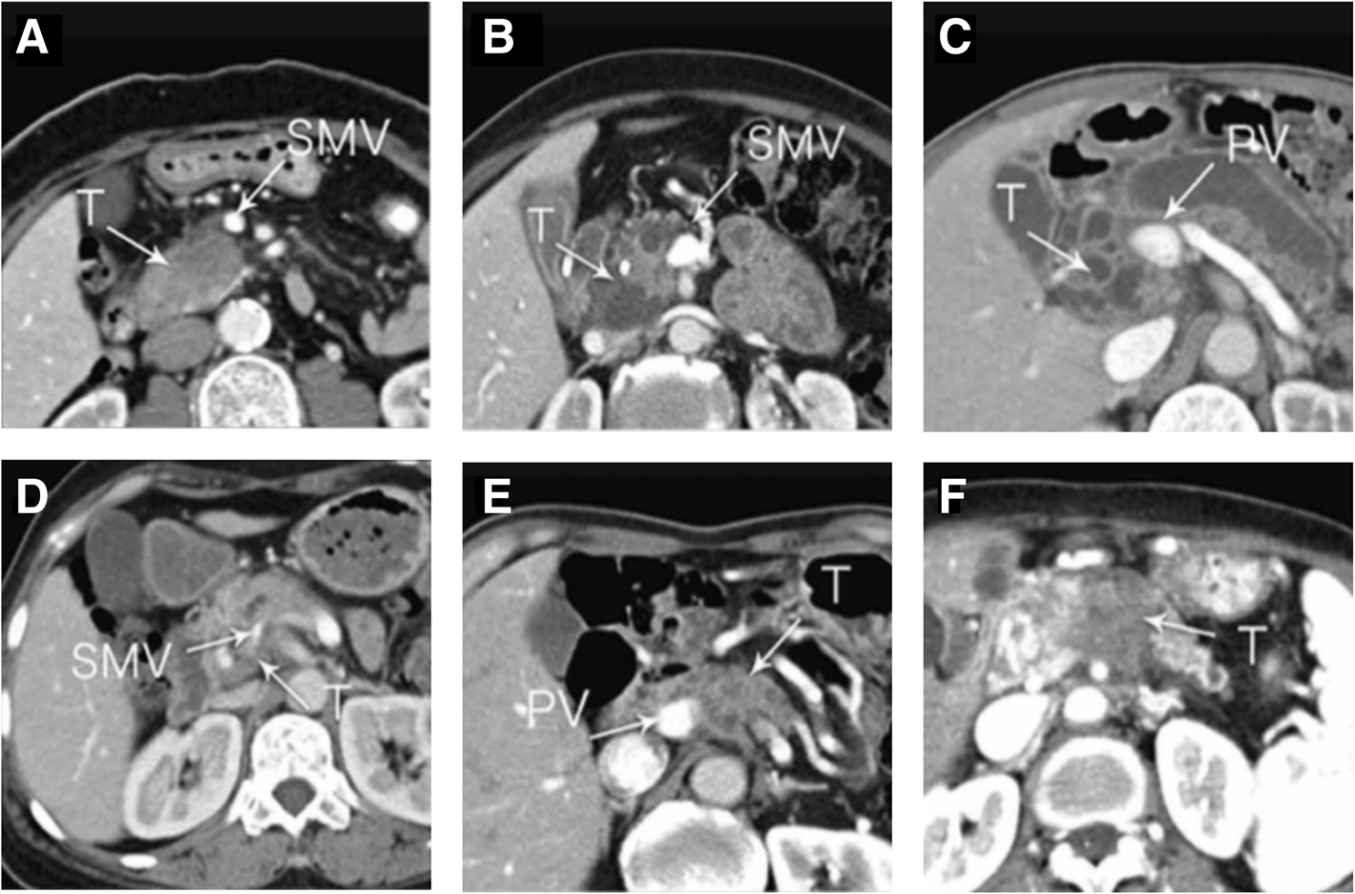
Venous contour abnormalities on CT. **a**, absent; **b**, tear drop; **c**, mild deformity; **d**, stenosis > 50%; **e**, tethering; **f**, obstruction

All images were retrospectively and independently analyzed by two radiologists (with 5 and 8 years of experience, respectively, in abdominal radiology). In case of discordance between the two radiologists, the images were evaluated by a third radiologist (with 25 years of experience in hepatobilliary and pancreatic radiology). The reviewers were only informed that the patients had pancreatic head cancer; they were blinded to all clinical data and outcomes. Tumor-associated venous contour abnormalities are difficult to describe. To standardize the radiologists’ reports, we classified the abnormalities into the following six types: absent, tear drop, mild deformity, stenosis > 50%, tethering, and obstruction (Fig. 1). The information of CT-equipment, imaging protocols and contrast injection protocols of the 4 institutions were listed in Additional file 1: Table S1.

### Pathological analysis

**Fig. 2:**
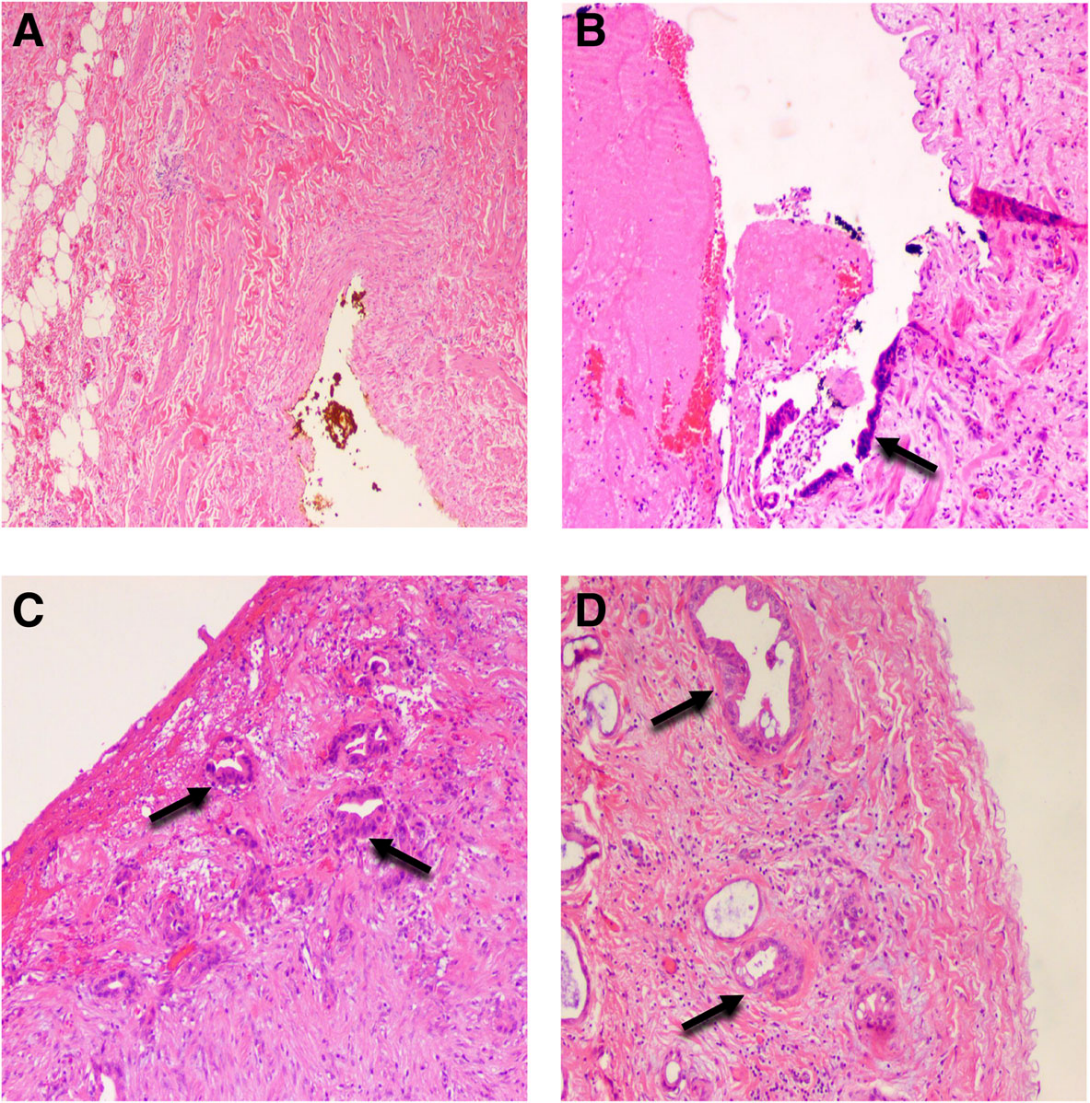
Pathological degree of carcinoma invasion of venous wall. **a**, Grade 0, no invasion (× 40); **b**, grade 1, tunica adventitia invasion (× 100); **c**, grade 2, tunica media invasion (× 100); **d**, grade 3, tunica intima invasion (× 100). Black arrow indicates cancer cells

Histopathology slides of all resected specimens were reviewed by pathologists with experience in pancreatic cancer, and venous wall involvement was graded as follows: grade 0 = no invasion; grade 1 = tunica adventitia invasion; grade 2 = tunica media invasion; or grade 3 = tunica intima invasion [11] (Fig. 2). Grade 1 or above was considered as pathologic vein invasion.

### Statistical analysis

All statistical analyses were performed with the SPSS 22.0 for Windows (IBM Corp., Armonk, NY, USA) and Prism 7 for Windows, version 7.00 (GraphPad Software, San Diego, CA, USA). Categorical variables were evaluated by using either the chi-square test or Fisher’s exact test. Continuous variables were compared using the Student’s *t*-test or the Mann–Whitney *U* test (for variables with non-normal distributions). All variables were incorporated into a univariate analysis and only those variables showing statistical significance (*P* less than 0.05) were evaluated by multivariate logistic analyses to identify the independent risk factors for peripancreatic vein invasion in patients with pancreatic head cancer. Survival analysis was with the Kaplan–Meier method; the log-rank test was used for comparisons between groups. Receiver operating characteristic (ROC) curves and the corresponding area under the curve (AUC) were used to assess how the predictive model performed on the test data.

The nomogram was formulated based on the results of multivariate logistic regression analysis, using the rms package of R, version 3.1.1 (http://www.r-project.org/). For constructing the nomogram, each of the independent predictors of venous invasion were assigned points proportionate to the value of its regression coefficient, the variable with the highest β coefficient being assigned 100 points. The total points were used to derive the predicted probability of venous involvement. The predictive performance of the nomogram was measured by calculating the concordance index (C-index) and by calibration with 500 bootstrap samples to decrease the overfit bias. *P* ≤ 0.05 was considered statistically significant for all tests.

## Results

### Patient characteristics

The study included 247 patients (144 male and 103 female) with mean age of 65 years. Patients from 3 centers were used as the training cohort (*n* = 181) while patients from the remaining center were used as the external validation cohort (*n* = 66). The proportion of the patients in these 2 groups was almost 3 to 1. The baseline characteristics of the patients did not differ significantly between these 2 groups (Table 1). At operation, tumor was adherent to the walls of the superior mesenteric and hepatic arteries in 9 patients, and so removal of the arterial sheaths was performed instead of arterial resection and reconstruction. This study did not include patients in whom preoperative radiological imaging revealed arterial invasion by tumor.

**Table 1:** Patient baseline characteristics by cohort

	Training cohort (*n* = 181)		Validation cohort (*n* = 66)		
	No. of patients	%	No. of patients	%	
Gender					0.657
Male	104	57.5	40	60.6	
Female	77	42.5	26	39.4	
Age, years					0.986
Median	65.0		64.0		
IQR	59.0–68.0		57.8–69.0		
ADL					0.781
Grade I	120	66.3	45	69.2	
> Grade I	61	33.7	21	31.8	
Jaundice					0.210
Yes	70	38.7	31	47.0	
No	111	61.3	35	53.0	
Pain					0.481
Yes	109	60.2	43	65.2	
No	72	39.8	23	34.8	
Weight loss					0.730
Yes	84	46.4	29	43.9	
No	97	53.6	37	56.1	
CA 19–9, U/mL					0.313
Median	280.4		439.1		
IQR	24.4–1614.1		53.9–2298.6		
TB, μmol/mL					0.956
Median	49.4		21.1		
IQR	11.2–151.1		11.1–182.9		
Albumin, g/L					0.349
Median	39.2		39.2		
IQR	36.1–41.8		36.6–43.7		
Tumor staging on CT					/
T1/T2	0	0	0	0	
T3	181	100	66	100	
T4	0	0	0	0	
Lymph node staging on CT					0.698
N0	80	44.2	31	47.0	
N1	101	55.8	35	53.0	
M staging on CT					/
M0	181	100	66	100	
M1	0	0	0	0	
TNM staging on CT					0.698
IA/IB	0	0	0	0	
IIA	80	44.2	31	47.0	
IIB	101	55.8	35	53.0	
III/IV	0	0	0	0	
Tumor size on CT, cm					0.701
Median	3.5		3.6		
IQR	2.8–4.6		2.8–4.44		
Length of tumor contact on CT, cm					0.849
Median	2.5		2.5		
IQR	2.0–3.0		2.0–3.0		
Pathologic venous wall invasion					0.868
Yes	152	84.0	56	84.8	
No	29	16.0	10	15.2	
Surgical margin					0.146
R0	163	90.1	55	83.3	
R1	18	9.9	11	16.7	

*ADL* activities of daily living, *CA 19–9* carbohydrate antigen 19–9, *TB* total bilirubin, *PV* portal vein, *SMV* superior mesenteric vein

### Survival analysis and risk factors for vein invasion

**Fig. 3:**
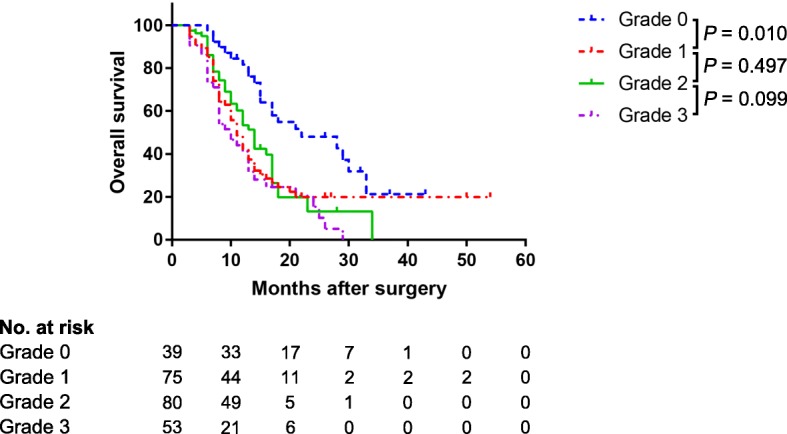
Cumulative survival rates according to pathological depth of vein invasion in the entire cohort

Figure 3 shows the cumulative survival rates in the entire cohort with different grades of histological vein invasion. Survival was significantly better in grade 0 than in grades 1, 2, and 3 (*P* <  0.001 for all). No significant difference in survival was noted between grades 1, 2, and 3.

In the training cohort, the univariate analysis showed that five factors—age, weight loss, length of tumor contact, circumferential vein involvement, and venous contour abnormalities — were significantly associated with vein invasion (Table 2). On multivariate analysis, however, only length of tumor contact (*P* = 0.031), circumferential vein involvement (*P* = 0.048), and venous contour abnormalities (*P* = 0.001) were independently associated with vein invasion (Table 2).

**Table 2:** Univariate and multivariate analyses of risk factors for histological venous invasion in the training cohort

Variables	Univariate analysis			Multivariate analysis		
	OR	95% CI	*P* value	OR	95% CI	*P* value
Gender (male/female)	1.836	0.825–4.088	0.137			
Age, years	1.076	1.028–1.125	**0.002**	1.046	0.983–1.113	0.156
ADL (grade I/>grade I)	1.732	0.695–4.316	0.238			
Jaundice (present/absent)	1.010	0.445–2.289	0.982			
Pain (present/absence)	1.281	0.575–2.854	0.545			
Weight loss (present/absent)	2.625	1.095–6.292	**0.030**	1.788	0.606–5.279	0.293
CA19–9, U/mL	1.000	1.000–1.000	0.272			
TB, μmol/L	0.998	0.994–1.002	0.242			
Albumin, g/L	1.014	0.924–1.114	0.763			
Lymph node staging on CT (N1/N0)	1.216	0.549–2.695	0.630			
Tumor size on CT, cm	1.010	0.790–1.293	0.934			
Length of tumor contact on CT, cm	3.425	1.940–6.047	**< 0.001**	2.067	1.070–3.995	**0.031**
Circumferential vein involvement on CT (>180°/90–180/≤ 90°)	4.443	2.297–8.595	**< 0.001**	2.207	1.008–4.832	**0.048**
Venous contour abnormalities on CT	3.004	0.022–4.463	**< 0.001**	2.121	1.368–3.289	**0.001**
Type of vessels involvement on CT (PV/SMV/PV + SMV)	1.132	0.671–1.910	0.641			

*ADL* activities of daily living, *CA 19–9* carbohydrate antigen 19–9, *TB* total bilirubin, *PV* portal vein, *SMV* superior mesenteric vein

### Predictive Nomogram construction and validation

**Fig. 4:**
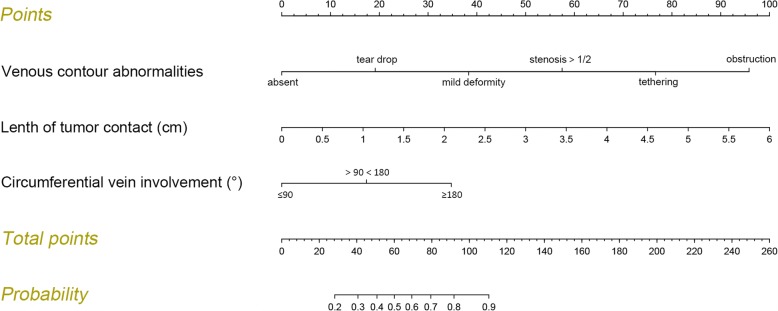
Predictive nomogram for assessing probability of peripancreatic vein invasion in patients with pancreatic head cancer. Points are assigned for each risk factor by drawing a line upward from the corresponding values to the “points” line. The total sum of points for the three risk factors is plotted on the “total sum points” line. A line is drawn down to read the corresponding predictions of vein invasion probabilities. For example, a patient with “dear drop” tumor-associated vascular abnormalities (18.5 points), with 5.5-cm tumor contact (92 points) and <90° circumferential vein involvement (0 point) will have a total score of 110.5, which corresponds to vein invasion probabilities of 89%

The nomogram for predicting probability of vein invasion was constructed using the three identified independent risk factors for vein invasion (Fig. 4). The points assigned to each factor were weighted by the ORs. The total score was used to calculate the probability of vein invasion. For example, a patient with suspected vein invasion had 2.5 cm tumor contact (41.5 points), with tear drop tumor-associated venous contour abnormalities (19 points) and > 180° circumferential vein involvement (35 points). Thus, the total score was 95.5, indicating 81% probability of vein invasion.

**Fig. 5:**
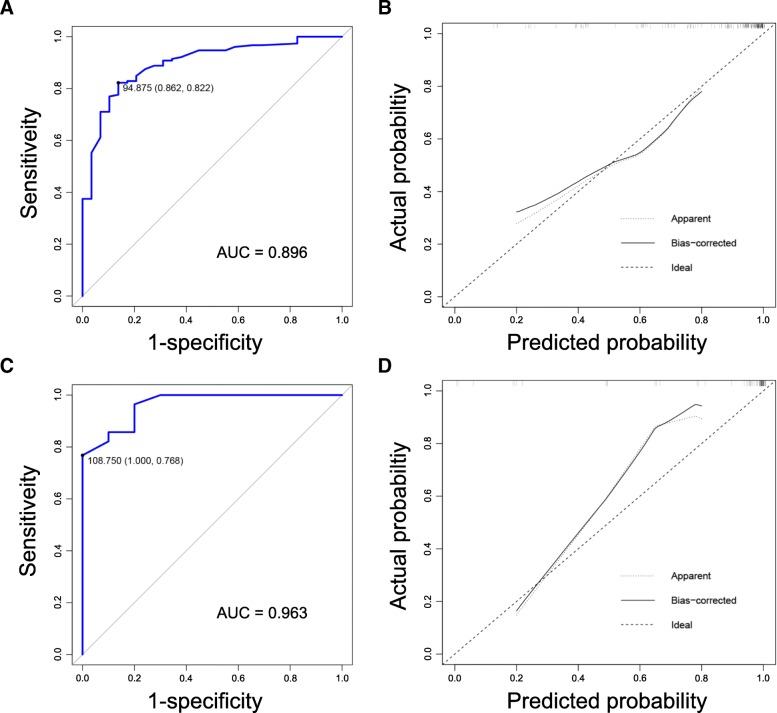
**a** Receiver operating characteristics curve for vein invasion in training cohort (using the nomogram values). The C-index is 0.896; **b** Calibration curves for comparison between predicted probability of vein invasion and observed vein invasion in training cohort (using the nomogram values); **c** Receiver operating characteristics curve for vein invasion in external validation cohort (using the nomogram values). The C-index is 0.963; **d** Calibration curves for comparison between predicted probability of vein invasion and observed vein invasion in training cohort (using the nomogram values)

The C-index of the nomogram for prediction of vein invasion was 0.896 for training cohort and 0.963 for validation cohort (Fig. 5a and c). In addition, the calibration curve revealed good agreement between estimation of vein invasion using our nomogram and the actual observation in both cohorts (Fig. 5b and d).

### Risk groups

**Fig. 6:**
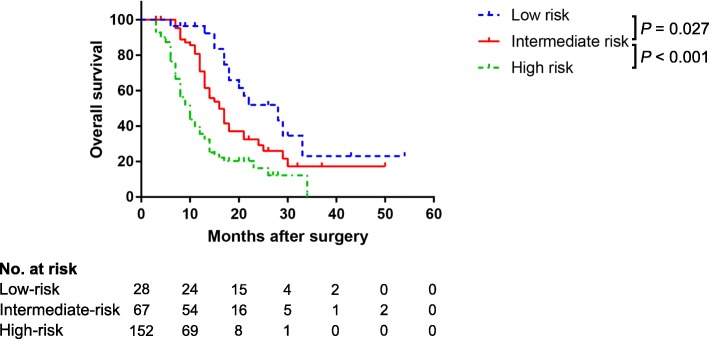
Cumulative survival rates in the different risk groups (the entire cohort)

Based on the risk estimated by the nomogram, the patients could be separated into three risk groups (Table 3): 1) a low-risk group (total points < 60 and predicted vein invasion rate < 50%), with a predicted mean risk of vein invasion of 32.69% (95% CI, 28.19–37.34); 2) an intermediate-risk group (total points 60–110.4 and predicted vein invasion rate 50–90%), with predicted mean risk of vein invasion of 76.15% (95% CI, 73.58–78.80); and 3) a high-risk group (total points > 110.4 and predicted vein invasion rate > 90%), with predicted mean risk of 92.56% vein invasion of (95% CI, 92.61–93.17). The observed vein invasion rates were slightly higher than the predicted mean risks. Significant differences in survival were noted between the risk groups (*P* <  0.001). Figure 6 shows the cumulative survival rates of patients in the three groups. Survival was significantly better in the low-risk and intermediate-risk groups than in the high-risk group (both *P* <  0.001). The difference between the low-risk and intermediate-risk groups was also significant (*P* = 0.027).

**Table 3:** Risk groups based on the predicted nomogram

Group	Total Points	Predicted Risk	Predicted Mean Risk (95% CI)	Observed Rate
Low-risk	< 60	< 50%	32.69% (28.19–37.34)	28.57% (8/28)
Intermediate-risk	60–110.4	50–90%	76.15% (73.58–78.80)	76.12% (51/67)
High-risk	> 110.4	> 90%	92.89% (92.61–93.17)	98.03% (149/152)

*CI* confidential interval

## Discussion

This study was performed to identify the risk factors of venous involvement in carcinoma head of pancreas patients and to use these to construct a nomogram for preoperative prediction of venous involvement. In general, our results were consistent with previous studies [16–22] showing that CT images can be used to assess the resectability of pancreatic cancer, although some possibility of misdiagnoses exists.

A positive surgical margin is a known poor prognostic factor [11]. Resection of invaded peripancreatic veins may be necessary to achieve a negative surgical margin during pancreaticoduodenectomy in patients with pancreatic head cancer [32]. However, efficient tools are still not available for reliable preoperative prediction of vein invasion. Nomograms, commonly used for decision-making in surgical oncology [27], can often provide accurate predictions of certain specified endpoints. To be practicable, however, the parameters included in the nomogram should be easily available and measurable [33].

Recently, in a consensus statement on the definition of BR-PDAC, in addition to arriving at an agreement on the anatomical factors to be included, the researchers also added biological and conditional host-related factors [31]. Accordingly, we attempted to include biological factors (e.g., CA-199, lymph node staging) and conditional host-related factors (e.g., activities of daily living) [34] —which were chosen to replace the Eastern Cooperative Oncology Group (ECOG) performance status—into our nomogram model. However, on univariate analysis none of these factors were significantly associated with venous invasion (Table 2). Further, in our review of relevant studies we found that previous authors have mainly focused on disease stage and prognosis of patients [35–39]. In our own previous unpublished research, also we found that factors like ages (*P* = 0.001) and lymph node metastasis (*P* = 0.001) are mainly associated with patients’ survival. Hence, we hold the opinion that peripancreatic vein invasion by pancreatic head cancer is mainly associated with anatomical factors. But biological and conditional host-related factors are more closely associated with disease progression.

In the latest National Comprehensive Center Network (NCCN) guidelines (version 2016/2017), only NAT is recommended for patients with BR-PDAC before tumor resection. However, imaging findings can be anamorphic after NAT, as viable tumor may be replaced by scar tissue. After NAT, the sensitivity and specificity of CT/MRI for detecting viable tumor are only 71 and 58%, respectively [40]. This discrepancy can affect the radiographic–histopathologic correlation and the performance of our nomogram. Therefore, only patients with pancreatic head cancer and suspected vein invasion who had undergone surgery but not received NAT in the past 5 years were included in the current study.

In this study some types of tumor-associated venous contour abnormalities, were found to be important indicators of venous invasion by tumor. In 2012, Nakao et al. [11] presented a radiographic typing using unilateral and bilateral narrowing of vessels as radiological evaluation indices; they suggested four types: type A (normal), type B (unilateral narrowing), type C (bilateral narrowing), and type D (complete obstruction, with collateral veins). Unfortunately, such this typing has weak ability to predict prognosis. But it was under debate whether venous walls with narrowing of > 180° should be considered unilateral narrowing or bilateral narrowing. Therefore, we included only the common vascular abnormalities such as absent, tear drop, mild deformity, stenosis > 50%, tethering and obstruction were included into current study.

Circumferential vein involvement has always been regarded as an important indicator of vein invasion [16–22]. However, in a recent study, Teramura et al. [20] reported that circumferential vein involvement has low diagnostic value and removed it from their proposed criteria. In an earlier unpublished study, we too failed to observe any significant effect of circumferential vein involvement on prognosis of patients with pancreatic head cancer after resectional surgery (*P* = 0.421), whereas anatomical factors were clearly associated with vein invasion. In the current study circumferential vein involvement was an independent risk factor for vein invasion (*P* = 0.048). In addition, length of tumor contact was also an independent risk factor for venous involvement. Tumor diameter on CT was also considered as a possible risk factor but was eliminated by univariate analysis (*P* = 0.934), once again confirming that vein invasion is not associated with disease progression.

The satisfactory performance of our nomogram in predicting vein invasion is reflected by the C-index of 0.896 and 0.963 in both cohorts, as well as by the calibration curves. By using the nomogram, we were able to classify pancreatic head cancer patients into three distinct risk groups. With the accumulation of clinical experience, specific management strategies can be provided according to the risks in the future. In the current study, survival analysis showed that prognosis is associated with the presence of pathological vein invasion but not with the depth of invasion (Fig. 3). This result also indicates that the accurate evaluation of vein invasion is important. Interestingly, survival varied between the three risk groups, being significantly higher in the low-risk and intermediate-risk groups than in the high-risk group (both *P* <  0.001). A significant difference in survival was also seen between the low-risk and intermediate-risk groups (*P* = 0.027).

This study has some limitations. First, although we included nearly 247 patients, the study sample is relatively small compared to some previous studies [11]. Second, in constructing the present nomogram, we did not consider the effect of tumor invasion of arterial structures such as the superior mesenteric artery, common hepatic artery, and celiac axis.

## Conclusion

Peripancreatic vein invasion in pancreatic head cancer patients is mainly associated with anatomical factors. Depth of wall invasion is not associated with prognosis. The proposed nomogram for preoperative prediction of vein invasion in patients with pancreatic head cancer showed satisfactory performance, with a C-index of 0.963 for external validation. The nomogram can be a convenient tool for facilitating decisions regarding the surgical approach.

## Additional file

Additional file 1: **Table S1.** Details of Examination Techniques. (DOCX 19 kb)

## Abbreviations

AJCC: American Joint Committee on Cancer
BR-PDAC: Borderline resectable pancreatic ductal adenocarcinoma
CI: Confidential interval
C-index: Concordance index
CT: Computed tomography
ECOG: Eastern Cooperative Oncology Group
NAT: Neoadjuvant therapy
NCCN: National Comprehensive Center Network
OR: Odds ratio
PSRR: Pancreaticoduodenectomy with PV/SMV resection and reconstruction
PV: Portal vein
SMV: Superior mesenteric vein
