# Supplementary material for: Preoperative prediction of peripancreatic vein invasion by pancreatic head cancer
Source: Cancer Imaging. 2018 Dec 10;18:49. doi: 10.1186/s40644-018-0179-z (PMC6288927; doi:10.1186/s40644-018-0179-z)
Supplement: Supplementary file 1 — Table S1. Details of Examination Techniques. (DOCX 19 kb) [file 40644_2018_179_MOESM1_ESM.docx]

**S-table 1. Details of Examination Techniques**

|  | Second Affiliated Hospital of Zhejiang University School of Medicine | Eastern Hepatobiliary Surgery Hospital | Tongji Hospital | Changhai Hospital |
| --- | --- | --- | --- | --- |
| CT-equipments | All examinations were performed with a multidetector CT scanner (Somatom Definition Flash Dual Source 64-row, Siemens Medical Systems, Erlangen, Germany). | All examinations were performed with a multidetector CT scanner (GE Lightspeed 64-row, GE Healthcare, USA). | All examinations were performed with a multidetector CT scanner (Somatom Definition Flash 64-row, Siemens Medical Systems, Erlangen, Germany). | All examinations were performed with a multidetector CT scanner (GE Lightspeed 64-row, GE Healthcare, USA). |
| Contrast injection protocols | Nonionic contrast medium (Iopamiro, 370 mg/ml, Bracco S.P.A.) 100ml was injected using a high-pressure injector at a rate of 3 mL/s. | Nonionic contrast medium (Omnipaque 350mg/ml; GE Healthcare, Milwaukee, WI, USA) 100ml was injected using a high-pressure injector at a rate of 3 mL/s. | Nonionic contrast medium (Ultravist 370 mg/mL; Bayer Healthcare, Berlin, Germany) 100ml was injected using a high-pressure injector at a rate of 3 mL/s. | Nonionic contrast medium (Omnipaque 350mg/ml; GE Healthcare, Milwaukee, WI, USA) 100ml was injected using a high-pressure injector at a rate of 3 mL/s. |
| Imaging protocols | The CT studies were performed with pre-contrast scans, arterial phase (25 s after injection), pancreatic phase (45 s after injection) and portal venous phase (65 s after injection). Scanning parameters were as follows: slice thickness, 0.6 mm; slice gap, 0.6 mm; 170 mAs, 120 kVp. Axial images and multiplanar reformats in the coronal plane at 3mm interval were reconstructed. MIP and CPR were used for vascular evaluation. | The CT studies were performed with pre-contrast scans, arterial phase (25 s after injection), pancreatic phase (45 s after injection) and portal venous phase (65 s after injection). Scanning parameters were as follows: slice thickness, 1.0 mm; slice gap, 1.0 mm; 146 mAs, 120 kVp. Axial images and multiplanar reformats in the coronal plane at 3mm interval were reconstructed. MIP and CPR were used for vascular evaluation. | The CT studies were performed with pre-contrast scans, arterial phase (25 s after injection), pancreatic phase (45 s after injection) and portal venous phase (65 s after injection). Scanning parameters were as follows: slice thickness, 1.0 mm; slice gap, 1.0 mm; 160 mAs, 120 kVp. Axial images and multiplanar reformats in the coronal plane at 3mm interval were reconstructed. MIP and CPR were used for vascular evaluation. | The CT studies were performed with pre-contrast scans, arterial phase (25 s after injection), pancreatic phase (45 s after injection) and portal venous phase (65 s after injection). Scanning parameters were as follows: slice thickness, 0.6 mm; slice gap, 0.6 mm; 160 mAs, 120 kVp. Axial images and multiplanar reformats in the coronal plane at 3mm interval were reconstructed. MIP and CPR were used for vascular evaluation. |
| MIP, maximum-intensity projections; CPR, curve planar reconstruction | | | | |
